# Physical activity patterns in type 1 diabetes in Spain: The SED1 study

**DOI:** 10.1186/s13102-023-00695-3

**Published:** 2023-07-26

**Authors:** F Gómez-Peralta, E Menéndez, S Conde, I Conget, A Novials, on behalf of SED and SED1 study investigators, Isabel Serrano Olmedo, Francisco Tinahones, Florentino Carral San Laureano, Martín López de la Torre, Alberto Moreno Carazo, Javier Acha, Orosia Bandrés, Lluís Masmiquel, Francisca Payeras, Ignacio Llorente Gómez, Juan Angel Hernández Bayo, Coral Montalbán, Daniel de Luis, Gonzalo Díaz-Soto, Antonio López-Guzmán, Estefania Santos-Mazo, Luz Mª López Jiménez, Visitacion Alvarez, Benito Blanco Samper, Ana Chico, Belen Dalama, Manuel Pérez-Maraver, Berta Soldevila, Ismael Capel Flores, Marta Hernández García, Wifredo Ricart, Ana Megia Colet, Elisenda Climent Biescas, Francisco Javier Ampudia-Blasco, Antonio Hernández-Mijares, Carlos Sánchez-Juan, Antonio Picó, José Ramón Domínguez Escribano, Carmiña Fajardo, Teresa Pedro, Pablo Abellán, Paolo Rossetti, Francisco M. Morales-Pérez, Fidel Enciso, Alfonso Soto González, Diego Bellido, Reyes Luna Cano, José Manuel García López, Víctor Manuel Andía, José Alfonso Arranz Martín, Sharona Azriel, Marta Botella Serrano, Miguel Brito Sanfiel, Alfonso Calle Pascual, Francisco Javier del Cañizo Gómez, Manuel Ángel Gargallo Fernández, Fátima Illán, Antonio M. Hernández Martínez, Lluis Forga Llenas, Sonia Gaztambide, Clara Rosario Fuentes Gómez, Amelia Oleaga, Mª Ángeles Martínez de Salinas, Juan Pedro López-Siguero, Ana Lucía Gómez-Gila, Alfonso María Lechuga Sancho, Marta Ferrer Lozano, Isolina Riaño Galán, María Caimari, Roque Cardona, María Clemente León, Gemma Carreras González, Francisco Javier Arroyo Diez, Paloma Cabanas Rodríguez, Belén Roldán, Noemí González Pérez del Villar, Purificación Ros Pérez, Itxaso Rica, Ignacio Diez López

**Affiliations:** 1 Endocrinology and Nutrition Unit, Hospital General de Segovia, Segovia, Spain; 2 Centro de Investigación Biomédica en Red de Diabetes y Enfermedades Metabólicas Asociadas (CIBERDEM), Barcelona, Spain; 3 Endocrinology and Nutrition Service, Hospital Universitario Central Asturias, Oviedo, Spain; 4 Centro de Salud de Barbastro, Huesca, Spain; 5 Endocrinology and Nutrition Unit, Hospital Clínic, Barcelona, Spain; 6 SED1 Study Investigators, Sociedad Española de Diabetes – SED, Madrid, Spain

**Keywords:** Diabetes Mellitus, Type 1, Insulin, Exercise, Hyperglycemia, Physical activity, Sports

## Abstract

**Aims:**

To describe the physical activity (PA) frequency and intensity in the Spanish type 1 diabetes mellitus (T1D) population and its association with their glycemic control.

**Methods:**

A cross-sectional observational study was carried out in 75 Spanish public hospitals (the SED1 study). T1D patients over 14years of age self-completed the International Physical Activity Questionnaire (IPAQ) to determine their level of exercise. The relationship between PA frequency and intensity in T1D patients and glycemic control and the diabetes therapeutic education received were analyzed.

**Results:**

A total of 592 patients were evaluable. A 6.8% of the sample performed light PA, 20.9% moderate and 72.3% vigorous. Estimated PA presented a high inter-individual variability. Men consumed more energy (METS) than women, these differences being more noticeable in vigorous METS (2865.80 in men vs 1352.12 in women). Women invested more min/week in the domestic and garden area (639.03 vs 344.39, *p* = 0,022). A correlation between glycemic control and the METs was not observed.

**Conclusions:**

The Spanish T1D population performed PA in a higher frequency and intensity than the general population. A relationship between PA and glycemic control couldn´t be shown. However, limitations of the study should be kept in mind to discard a long-term positive influence.

**Supplementary Information:**

The online version contains supplementary material available at 10.1186/s13102-023-00695-3.

## Introduction

Type 1 diabetes mellitus (T1D) is an autoimmune disease reducing insulin production and therefore requires daily insulin administration [1]. The prevalence and incidence of T1D is increasing worldwide, according to recent studies [2]. A recently published meta-analysis reported that T1D incidence was 15 per 100,000 people per year, and the prevalence was 9.5 per 10,000 people [3].

The American Diabetes Association (ADA) states that physical activity (PA) is a core element for metabolic management in individuals with diabetes mellitus (DM) [4]. It has been described that aerobic PA increases cardiorespiratory fitness, decreases insulin resistance, and improves lipid levels and endothelial function in T1D [4, 5]. However, the energy-consuming effect of exercise can cause hypoglycemia during or after practice [6]. Then, adjusting the insulin and nutritional treatment to PA and sports can be challenging for healthcare providers and people with T1D. Factors related to the intensity and duration of PA, individual characteristics, and the insulin regimen can influence the benefits and risks [7].

Most of the studies focused on PA performed in people with diabetes agree on their beneficial effects [4, 5]. The metabolic effects should allow the T1D person to improve plasma glucose levels [5]. Apart from improving blood glucose levels, PA works by increasing respiratory capacity, stimulating muscle blood circulation (favoring the heart), reducing and maintaining an adequate weight, releasing endorphins producing a feeling of well-being, among other advantageous outcomes [8–10]. PA has these positive effects at certain intensity and volume, better known as physical exercise. Thus, the ADA and also different international guidelines recommend performing regular PA, however the intensity and type of PA should be adapted according to the patient’s health status [4, 11, 12].

To maintain glycemic concentrations during and after PA in T1D, regular blood glucose checking or trends and values evaluation using continuous glucose monitoring (CGM) and the management of additional carbohydrates are crucial [13].

There are various questionnaires and methods to measure PA. The International Physical Activity Questionnaire (IPAQ) is an internationally accepted, validated, and reliable tool. It allows to measure PA in different populations between 15 and 69 years [14, 15]. At present, there is a limited amount of scientific evidence assessing the impact of PA on glycemic control in patients with T1D. This study aims to describe the PA frequency and intensity in the Spanish T1D population, its association with their glycemic control, and how demographic factors and pharmacological treatment influence this relationship.

## Materials and methods

### Study design

The already published SED1 study was a multicentric cross-sectional observational study including a representative sample of the T1D Spanish population treated in the consultations of endocrinology specialists from 75 public hospitals in Spain [16]. Patients had to meet the following criteria: patients diagnosed with T1D, with a medical record at the site, with at least two HbA1c values available in the study visit and written informed consent. Patients with a diagnosis of type 2 diabetes mellitus (T2D) and patients with a history of pancreas and/or islet cell transplantation were excluded.

The sample size had to be sufficient to describe the sociodemographic and clinical profile of patients with T1D. As it was based on a description of the study population, the minimum sample size required to estimate any dichotomous variables that might appear with a probability of 0.50 (value requiring the maximum sample size) was calculated. Assuming a total Spanish population size of 46,454,535 inhabitants (according to Instituto Nacional de Estadística [Spanish National Statistics Institute],INE) data from 01/07/2016), with a prevalence of T1D of 0.1%, the population of patients with T1D was estimated at 46,455. To estimate dichotomous variables with a p of 0.5, an accuracy of 0.04 and a level of significance of 0.05, a sample for the complete SED1 study of at least 594 adult patients with T1D had to be included.

The protocol included an assessment of the duration and frequency of PA through self-administered IPAQ, completed by included patients during the unique study visit.

This study was performed in accordance with the principles stated in the Declaration of Helsinki and approval was granted for the study by the Spanish Agency of Medicines and Medical Devices (AEMPS) and the ethics committe of the Hospital General de Segovia (Spain) (*Comité de Ética de la Investigación con medicamentos -CEIm- del Área de Salud de Segovia*) and the rest of participant hospitals. All participants provided written informed consent before entering the study.

### Physical activity measurement: The IPAQ questionnaire

The IPAQ questionnaire is a standardized tool, validated in the Spanish population, developed to measure health-related PA [15] in people over 15 years old. The long IPAQ version comprises a set of four questionnaires with five activity domains requested independently. The long version is recommended in research studies as it provides more detailed information. It is a 27 item questionnaire that refers to the time the patient has been physically active in the last seven days.

The final score is the sum of the duration (in minutes) and frequency (in days) for all types of activity. Results can be reported in categories (low activity levels, moderate activity levels or high activity levels) or as a continuous variable (metabolic equivalent -MET- minutes a week). One MET is what a person expends when at rest [17]. The IPAQ quantifies the volume of physical activity by assigning defined energy requirements in METs to each intensity level. Thus, the results were obtained in MET-minutes by multiplying the METs assigned to an activity (walk × 3.3, moderate activity × 4, vigorous activity × 8) by the minutes in which it had been carried out and in MET minutes a week multiplying it in turn by the number of days per week.

The scoring classifies every person to one of three levels depending on the PA:

- Category 1: Low: this is the lowest level of PA. Those individuals who do not meet the criteria for categories 2 or 3 are considered low/inactive.
- Category 2: Moderate: Any one of the following three criteria:
  • three or more days of vigorous activity of at least 20 minutes per day OR
  • five or more days of moderate-intensity activity or walking of at least 30 minutes per day OR
  • five or more days of any combination of walking, moderate-intensity, or vigorous intensity activities achieving a minimum of at least 600 MET minutes a week.
- Category 3: High: Any one of the following two criteria:
  • vigorous activity on at least three days and accumulating at least 1500 MET minutes a week OR
  • seven or more days of any combination of walking, moderate-intensity or vigorous intensity activities, achieving a minimum of at least 3000 MET minutes a week.

Those who did not reach a minimum of minutes and/or days per week of vigorous, moderate or walking activities met the criteria for low PA levels and were thus considered sedentary individuals. Conversely, those individuals meeting the criteria for high or moderate PA categories were identified as active individuals.

### Endpoints

The primary endpoint was to describe the PA frequency and intensity in T1D patients.

Other secondary endpoints were the description of the relationship between PA and the glycemic control (glycated haemoglobin A1c -HbA1c-, pre- and postprandial glucose profiles and hypoglycemia), clinical characteristics, sociodemographic factors (mean age, level of education etc.), T1D duration, type of treatment (method of administration and dose of insulin), hospital admissions and visits to healthcare providers in the last 12months. The diabetes therapeutic education received and its effects on the quality of diabetes self-management were analyzed using the following three variables: use of an insulin/carbohydrate ratio, use carbohydrate count, and calculation of an insulin sensitivity factor. All were also grouped as "Use of advanced treatment with insulin” for a composite variable describing therapeutic education level.

The 10-year cardiovascular risk prediction of non-fatal and fatal cardiovascular (CV) disease (ischemic heart disease, stroke, peripheral vascular disease) according to the Steno Type 1 Risk Engine was calculated [18].

### Statistical analysis

Continuous variables were described by the number of patients with valid observations, mean, and standard deviation (SD). Categorical variables were described by the number and percentages of patients per response category.

Linear regressions were used to identify factors (age, sex, body mass index -BMI-, time since T1D diagnosis, educational level, smoking status, alcohol intake, use of advanced treatment) associated with PA. Later, univariate/multivariable logistic and linear regression models were used to evaluate the relationship between METs (independent variable) and clinical characteristics of T1D (dependent variables). Age, sex, BMI and time since diagnosis were considered confounding factors in multivariable models (except CV risk, where the variables used to compute CV risk index were excluded). All models have been carried out for each intensity of PA (low, moderate, high and total).

A *p*-value of < 0.05 was considered statistically significant. Statistical analyses were generated using SAS software, version 7.15 Enterprise Guide.

## Results

### Population

A total population of 647 patients were eligible in the SED1 study and 592 T1D participants who answered the IPAQ questionnaire were included in this subanalysis.

Sociodemographic and clinical characteristics are described in Table 1. The mean age population was 38.8 ± 12.8years, more than 80% of the T1D population had secondary/university studies, and 22% were current smokers. A 13.1% of the population presented obesity (BMI ≥ 30kg/m^2^). The mean time since the T1D diagnosis was 19.1 ± 11.7years, and the mean HbA1c was 7.6 ± 1.1%[60 ± 12mmol/mol].

**Table 1:** Sociodemographic and clinical characteristics of the population

	**Total Population (*N* = 592)**
**Age** (years), mean (SD)	38.8 (12.8)
**Range of ages** (years), (%)	
15–17	17 (2,9%)
18–25	92 (15,5%)
26–49	363 (61.3%)
50–60	94 (15.9%)
> 60	26 (4.4%)
**Gender**, women, n (%)	330 (55.7%)
**Ethnicity**	
African	3 (0.5%)
Asian / Oriental	1 (0.2%)
Caucasian	576 (97.5%)
Hispanic—American	11 (1.9%)
**Educational Level**, n (%)	
Without studies	1 (0.2%)
Primary studies	87 (15.6%)
secondary studies	224 (40.1%)
University Study / Similar	228 (40.9%)
Student	18 (3.2%)
**Smoking Status**, n (%)	
Current Smoker	127 (22.0%)
Ex-smoker (> 6 months without smoking)	78 (13.5%)
Non-smoker	373 (64.5%)
**Alcohol Intake**, n (%)	
Never	306 (55.4%)
< 1 day/week	165 (29.9%)
1–6 day/week	64 (11.6%)
Every days	17 (3.1%)
**BMI (kg/m2),** mean (SD)	25.2 (4.2)
**BMI Category,** (%)	
< 18 Underweight	8 (1.4%)
18.5–24.9 Normal	326 (55.5%)
**SBP** (mmHg), mean (SD)	124.2 (15.8)
**DBP** (mmHg), mean (SD)	73.3 (9.1)
**Waist circumference** (cm), mean (SD)	87.0 (13.0)
**Time since T1D diagnosis** (years), mean (SD)	19.1 (11.7)
**HbA1c,** mean (SD)	
%	7.6 (1.1)
mmol/mol	60.0 ±12.0 mmol/mol
**Pre-prandial Glucose**^a^, mean (SD)	
mg/dL	145.2 (46.0)
mmol/L	8.1 (2.6)
**Post-prandial Glucose**^b^, mean (SD)	
mg/dL	163.1 (49.9)
mmol/L	9.1 (2.8)
**CV Risk** (%), mean (SD)	9.3 (8.5)

*SBP* systolic blood pressure, *DBP* diastolic blood pressure, *SD* Standard deviation, *CV Risk* 10-year prediction of non-fatal and fatal cardiovascular disease (ischemic heart disease, stroke, peripheral vascular disease) risk according to the Steno Type 1 Risk Engine [18]

^a^Pre-prandial glucose: mean of the glucose values before the three main meals

^b^Post-prandial glucose: mean of the glucose values two hours after the three main meals;

Basal-bolus was the most frequent method of insulin administration in our population (77.7%), followed by continuous subcutaneous insulin infusion (CSII) (20.8%) (Supplementary table 1S). The mean total daily insulin dose was 0.6 ± 0.3 UI/Kg/day. A 77.5% of the patients performed self-monitoring of blood glucose (SMBG), while 22.5% used some CGM system.

### Distribution of physical activity intensities

According to the IPAQ categorical score, 6.8% of the sample performed light PA, 20.9% moderate and 72.3% vigorous PA. Estimated PA reported through IPAQ (METs) presented a high inter-individual variability, with a mean (SD) of 6478.97 (5206.94).

Regarding the distribution of the total METs, 31.2% corresponds to vigorous activities, 38.8% to moderate and 30% to walking (Table 2). When relating the METs to the characteristics of the patients, statistically significant differences were observed by sex and age, not being observed by BMI. Men generally consumed more energy (METS) than women, being these differences more noticeable in vigorous METS (2865.80 in men vs 1352.12 in women). Total and vigorous PA steadily reduces with age over 18years old. However, this association is lost when p is adjusted by sex, BMI and or time since the diagnosis of T1D.

**Table 2:** IPAQ results in MET minutes a week stratified by gender, age, and BMI

	**Total Walking MET minutes a week**	**Total Moderate MET minutes a week**	**Total Vigorous MET minutes a week**	**Total MET minutes a week**
**Total**	1945.59 (1524.55)	2511.35 (1951.32)	2022.03 (3139.74)	6478.97 (5206.94)
**Sex**				
Man	2018.08 (1581.76)	2404.89 (2024.75)	2865.80 (3472.00)	7288.76 (5721.41)
Woman	1888.04 (1477.44)	2595.88 (1889.82)	1352.12 (2669.84)	5836.04 (4668.78)
* p*-value	0.4578	**0.0023**	**0.0118**	0.5814
p-adjusted	0.255	0.330	** < 0.001**	**0.001**
**Age**				
15–17	1686.88 (1602.87)	1252.94 (1592.69)	2145.88 (2629.71)	5085.71 (4725.98)
18–25	1844.05 (1536.93)	2001.52 (1913.59)	2180.00 (3121.39)	6025.58 (5062.77)
26–49	1923.06 (1524.49)	2640.77 (1914.91)	2132.56 (3235.21)	6696.40 (5367.80)
50–60	2067.94 (1497.37)	2705.96 (2064.76)	1782.13 (3150.88)	6556.03 (5203.55)
> 60	2346.17 (1547.74)	2627.69 (1916.26)	706.15 (1538.37)	5680.02 (3456.45)
* p*-value	0.5103	0.1282	** < 0.0001**	**0.0081**
p-adjusted	0.090	0.124	0.462	0.520
**BMI**				
< 18.5	2479.13 (1739.36)	2730.00 (1892.87)	540.00 (788.20)	5749.13 (2549.11)
18.5–24.9	1933.44 (1507.34)	2406.20 (1903.08)	1881.96 (2884.25)	6221.60 (5012.91)
25–26.9	2135.28 (1638.60)	2794.47 (1915.66)	2669.36 (3737.85)	7599.11 (5806.86)
27–29.9	1906.51 (1455.94)	2169.51 (1932.83)	1756.10 (2887.20)	5832.12 (4761.39)
> = 30	1750.07 (1538.97)	2922.08 (2143.67)	2374.55 (3713.57)	7046.69 (5824.26)
* p*-value	0.5413	0.1017	0.4230	0.2685
p-adjusted	0.268	0.301	0.123	0.333

*p*-value without being adjusted

*p*-adjusted by age (continuous variable), sex, BMI (continuous variable) and T1D duration, adjusted for the other variables (it has been adjusted excluding the observed variable)

### IPAQ results on physical activity frequency

**Fig. 1:**
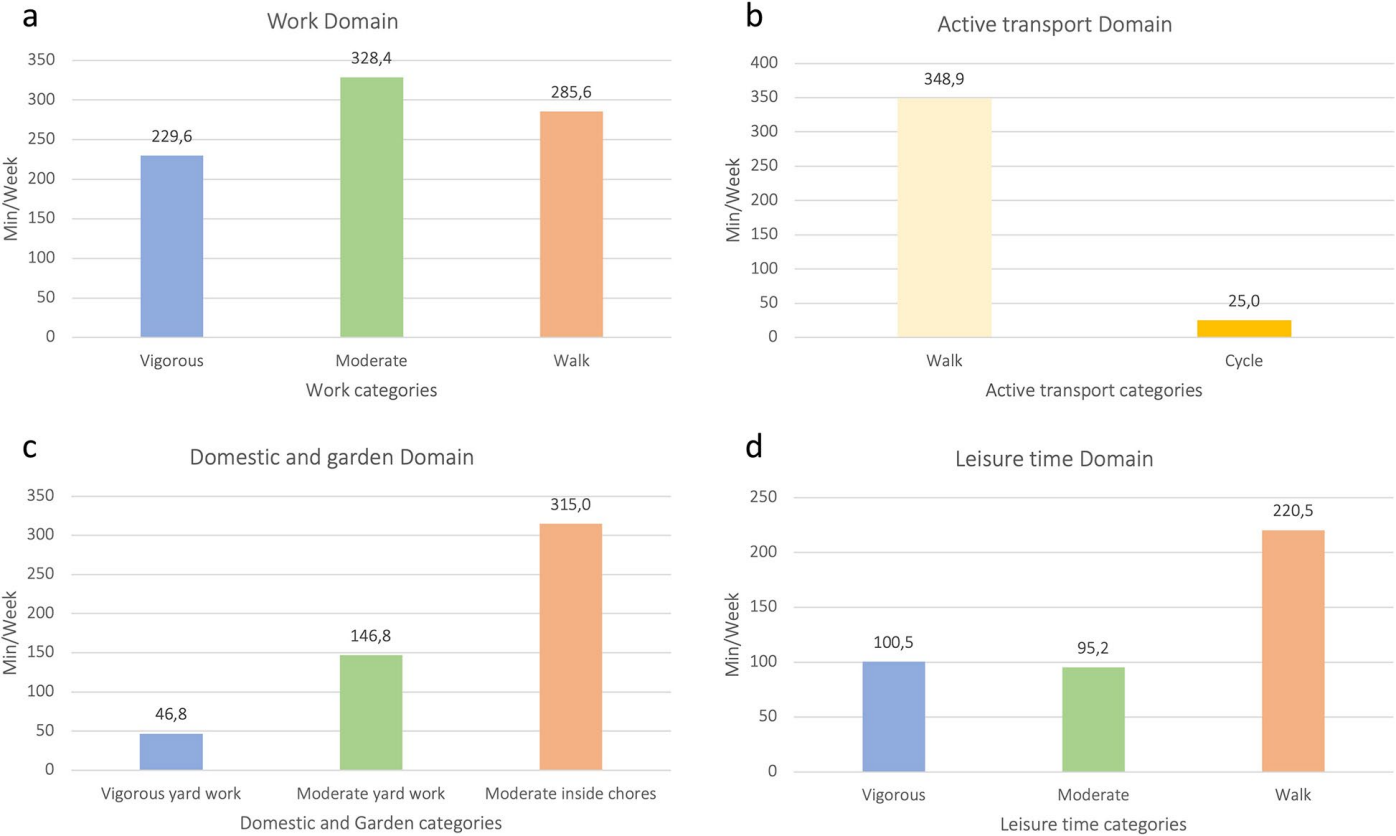
Time spent in each IPAQ domain

Figure 1 shows the time that participants spent on all the domains that the IPAQ covers- work, active transport, domestic and garden, and leisure time domain-, according to the intensity of the activity.

With regards to the work domain, 38.9% of this time corresponded to moderate physical activities (328.4 MET minutes a week, while 38.9% was devoted to vigorous activities (229.6min per week) and 33.9% to walking (Fig. 1a). If we observe time invested in the transport domain (Fig. 1b), we can appreciate that individuals spent 93.3% of time walking (348.9min per week), using 25min per week on cycling. The highest mean time spent in domestic and garden activities (Fig. 1c) corresponds to the moderate and inside chores category (315min per week), followed by moderate (146.8min per week) and vigorous garden work activities (46.8min per week). Finally, concerning leisure time, results indicate that most time is invested in walking (220.5min per week), whereas similar time is used for moderate and vigorous activities (95.2 and 100.5min per week, respectively) (Fig. 1d).

Results from the IPAQ reported that the mean (SD) for the sedentary time was 1790.28 (1219.19) min per week.

### Types of physical activity

Regarding the type of PA performed per week, work was the domain associated with the highest minutes per week (843.6min per week), followed by the domestic and the garden domain (508.6min per week), the leisure-time (416.2min per week) and the transport domain (373.9min per week).

**Fig. 2:**
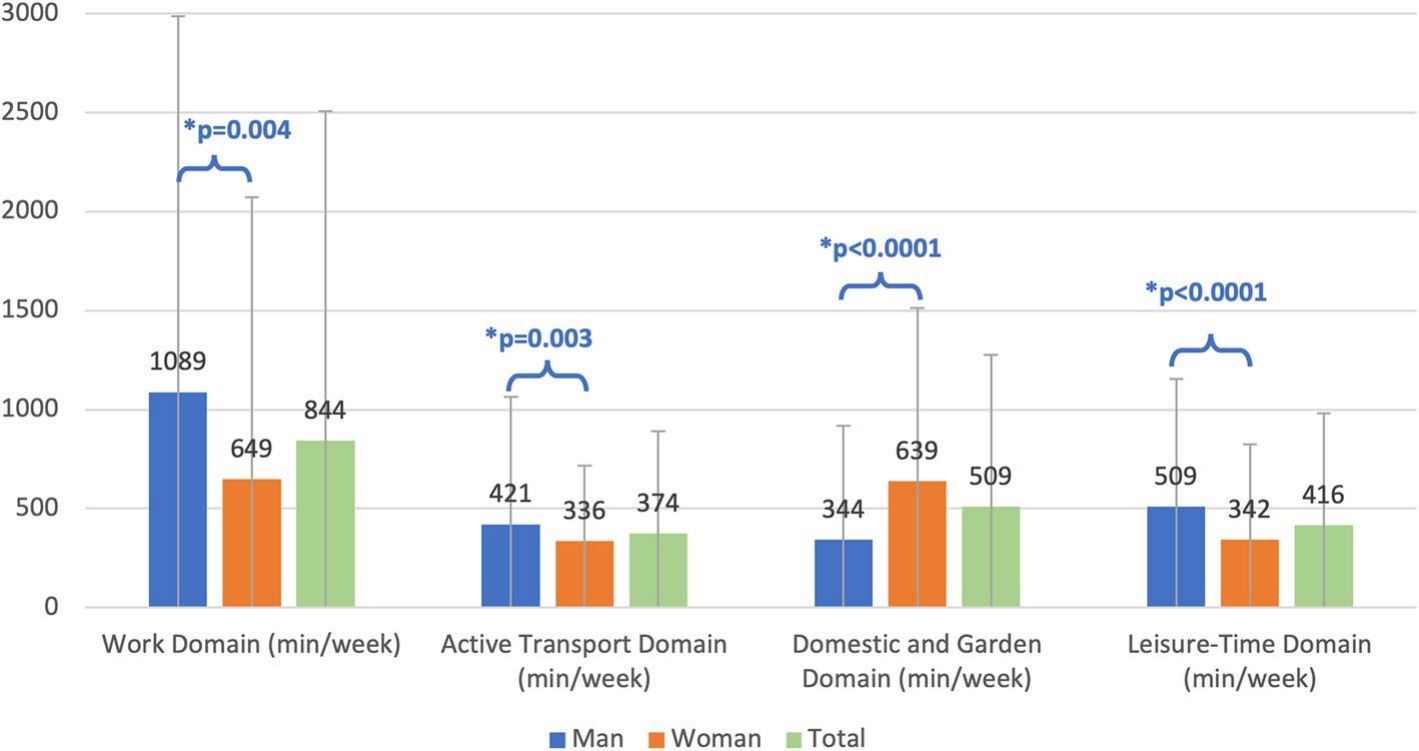
IPAQ Domains in min/week stratified by gender

Results from the IPAQ questionnaire were presented by gender (Table 3 and Fig. 2). They showed significant differences in min/week invested in each of the domains that IPAQ covers (*p* = 0,004 for Work, p = 0,030 for active transport and *p* < 0,001 for domestic and garden and leisure-time domains). It seems that males invest more min per week than females in all the domains, except for domestic and garden area, where females spent more time (639.03 vs 344,39, *p* = 0,022).

**Table 3:** IPAQ results in min/week stratified by gender, age, and BMI

	**Work Domain (min/week)**	**Active Transport Domain (min/week)**	**Domestic and Garden Domain (min/week)**	**Leisure-Time Domain (min/week)**
**Total**	843.56 (1664.95)	373.92 (516.02)	508.63 (769.69)	416.16 (567.14)
**Sex**				
Man	1088.85 (1899.00)	421.42 (643.84)	344.39 (576.35)	509.45 (646.28)
Woman	648.82 (1425.75)	336.20 (382.42)	639.03 (873.01)	342.09 (483.75)
* p*-value	**0.0014**	**0.0458**	** < 0.0001**	**0.0003**
p-adjusted	**0.004**	**0.030**	** < 0.001**	** < 0.001**
**Age**				
15–17	266.47 (872.70)	342.94 (428.64)	261.47 (544.87)	641.76 (771.08)
18–25	792.61 (1562.39)	336.09 (487.81)	277.66 (524.72)	375.76 (417.86)
26–49	926.60 (1732.72)	370.79 (525.63)	521.18 (715.18)	408.39 (593.31)
50–60	862.29 (1765.78)	419.31 (551.85)	635.00 (736.72)	408.88 (550.77)
> 60	174.23 (531.53)	407.50 (403.05)	855.38 (1721.09)	546.35 (550.16)
* p*-value	0.1231	0.8440	**0.0012**	0.3303
p-adjusted	0.778	0.133	**0.022**	0.701
**BMI**				
< 18.5	372.50 (686.77)	353.75 (234.33)	820.00 (952.71)	453.75 (413.66)
18.5–24.9	726.83 (1534.62)	379.37 (495.73)	464.05 (627.31)	426.93 (577.17)
25–26.9	1082.71 (1970.90)	459.18 (627.77)	535.43 (669.66)	558.19 (755.28)
27–29.9	800.37 (1634.42)	340.06 (542.95)	388.35 (546.87)	356.88 (410.55)
≥ 30	1172.21 (1878.30)	277.21 (436.24)	734.61 (1337.90)	271.10 (354.79)
* p*-value	0.1022	**0.0294**	0.3764	**0.0105**
p-adjusted	**0.0207**	0.118	0.294	**0.049**

*p*-value without being adjusted

When we examined the effect that age may have had on individuals' time on PA, we detected significant differences just in the domestic and garden domain, where the time increased as people got older (*p* = 0,022) (see Table 3).

An association of BMI with work (*p* = 0,027) and leisure-time domains (*p* = 0,049) is observed, even adjusting by sex, age, and T1D duration. As it could be expected, the time consumed in the leisure-time domain is lower in the lowest (< 18.5kg/m^2^) and in the highest BMI (over 27kg/m^2^ categories), the underweight and overweight T1D individuals. However, an unexpectedly high consumed time in the work domain is reported by obese (BMI > 30kg/m^2^) T1D participants (see Table 3).

Time from diagnosis was not correlated to the minutes per week invested by the participants in PA. Following this pattern, no significant differences were observed between the analyzed characteristics in sedentary time.

### Physical activity and glycemic control

**Fig. 3:**
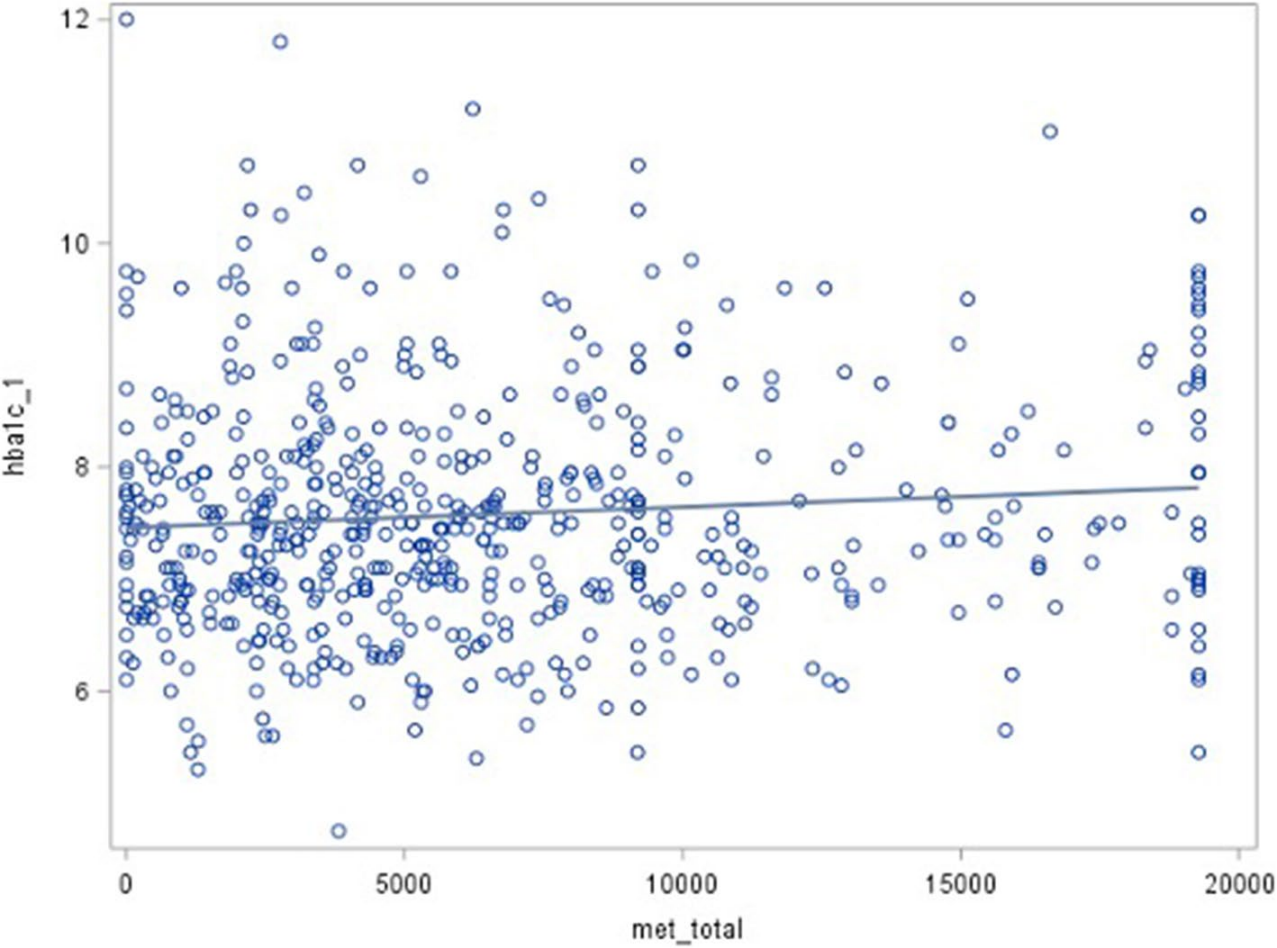
Scatter plot representing total MET and HbA1c level

Results from the analysis did not show a correlation between glucose control defined by HbA1c (Supplementary, Table 2S and Fig. 3). Even more, a slightly greater HbA1c was associated with higher moderate and total PA intensities (Pearson correlation coefficients: 0.11587; *p* = 0.0048, and 0.08938; *p* = 0.0298, respectively).

Any statistical correlation was observed between PA and pre or postprandial glycemia, therapeutic education, the types of insulin therapies, hypoglycemia and complications of the disease in patients diagnosed with T1D with the METs performed by the participants. (Supplementary table 2S).

### Physical activity and predicted cardiovascular risk

The energy (METS) consumed in vigorous PA showed a negative bivariable association with the predicted 10-year cardiovascular risk according to the Steno Type 1 Risk Engine (*p* = 0,0392). This association is lost when p is adjusted by age (continuous variable), sex, BMI (continuous variable) and T1D duration.

### Physical activity and T1D treatment, sociodemographic, or clinical variables

The analysis of therapeutic education related variables (use of an insulin/carbohydrate ratio, use carbohydrate count and calculation of a sensitivity factor), the types of insulin therapies (method of administration and dose of insulin), clinical characteristics, sociodemographic factors (level of education etc.), T1D duration, and burden of the disease (hospital admissions and visits to healthcare providers in the last 12months) did not show a statistical correlation. (Supplementary table 2S).

## Discussion

Our study results showed that 6.8% of the Spanish T1D population performed light PA, 20.9% moderate and 72.3% vigorous PA. These results described a higher performed PA intensity in T1D people than in the general Spanish population according to the Spanish Ministry of Health data using the same IPAQ questionnaire and similar population features (adults 15–69 y, mean age = 43 y, *n* = 17,777): 35,3% light PA, 40,4% moderate and 24,3%, vigorous PA [19]. This is an encouraging result, pointing to a higher motivation towards a healthy lifestyle in the T1D population, probably related to the effect of therapeutic education displayed in the last decades [20].

Regarding the distribution of the total PA performed, 31.2% corresponds to vigorous activities, 38.8% to moderate and 30% to walking. Guidelines [4] recommend that adults with T1D should engage in 150min or more of moderate-to vigorous-intensity physical activity, spread over at least three days/week, with no more than two consecutive days without activity. Attending to our results and attending exclusively to the leisure time, results indicate that the time used for moderate and vigorous activities (95.2 and 100.5min per week, respectively) could indicate that, in general, people with T1D are accomplishing this recommendation. However, reported PA presented a high inter-individual variability suggesting that an individual approach to the PA evaluation and management in this population is needed.

The data obtained regarding the vigorous activities on leisure time could indicate that the time used for sports (100.5min per week) is reduced compared with moderate and light PA (315,7min per week). For this reason, when clinical recommendations and guidelines are designed, it should be kept in mind to avoide focusing exclusively on sports or high-intensity practice.

Attending to our results of total MET minutes a week stratified by age, we observe that middle-age T1D people consumed more energy in PA than younger or older participants. Additionally, in the domestic and garden domain, the time spent in PA increased as people got older. Finn et al. [21] data in 2021 indicated that, when stratified by age, the amount of moderate or vigorous PA decreased with age. Guidelines state that both young and adults with T1D can benefit from being physically active, and thus, activity should be recommended to all [4, 11].

Our results showed that men generally spend more energy (METs) than women, except for the domestic and garden domain. These differences were more noticeable in vigorous METs (2865.80 in men vs 1352.12 in women). In agreement with these results, a Spanish population-based study reported a higher proportion of women performing lower energy expenditure in physical activities [22]. The McCarthy et al. study (2017), showed that more women were in the sedentary category compared to men in adults with type 1 diabetes [23]. Another recent publication demonstrated that more men than women met the PA guidelines [21]. However, the time spent by women on the domestic and garden domain was clearly higher than in men (639.03 vs 344.39min/week, *p* < 0.001). It supports the persistence of the traditional sex roles also in this population.

A correlation between overall glycemic control (HbA1c) and the METs performed by the participants was not observed. In a systematic review in people with T1D [9], reductions of HbA1c by exercise intervention were observed. However, another literature review [5] reported that studies investigating the effect of PA on glycemic control in T1D have largely failed to demonstrate a benefit. It should be mentioned that the Spanish RECORD guideline on clinical recommendations for the practice of sports in people with diabetes mellitus have reported that there is not sufficient evidence to conclude that sustained exercise consistently improves HbA1c levels in adults with T1D [12]. The acute effect of PA on glycemic control after exercise remains unclear, probably depending on the duration, type and the insulin treatment management [24]. However, PA should be recommended due to its other benefits to the cardiovascular system. In this line, the American Diabetes Association (ADA) and the European Association for the Study of Diabetes (EASD) [25], even recognizing gaps in evidence on independent effects of PA on beta-cell function and HbA1c, included in its recent guideline on T1D management that people with T1D should be encouraged to engage in exercise because of improved fitness, increased insulin sensitivity, reduced insulin requirement, improved cardiovascular, and decreased mortality. Additionally, a more comprehensive description of glycemic control, including glycemic variability and glucometrics derived from continuous glucose monitoring, could be more precise to describe the effects of PA on glycemic dynamics in T1D people [13, 24].

Several studies have reported outcomes where PA seems to be beneficial in people with T1D, playing, for instance, an important role in the prevention of cardiovascular disease [5, 9, 26]. It is worth mentioning a longitudinal study [27] that assessed the benefit of cycling in persons with diabetes whose results showed that cycling was associated with at least a 24% lower all-cause mortality when compared with noncyclists, independent of other physical activities and possible confounders. In Spain, low PA is rising, especially among women [22]. In 2019, a meta-analysis revealed that exercise training might result in positive changes in biological cardiovascular risk factors, including aerobic fitness, HbA1c, insulin dosage, and lipids in persons living with T1D [9]. However, in our study only the energy (METS) consumed in vigorous PA could positively affect the predicted 10-year cardiovascular risk.

Finally, we did not observe a correlation between the rest of the studied variables (therapeutic education, the types of insulin therapies, hypoglycemia and complications of the disease in patients diagnosed with T1D) with the PA performed by the participants.

One strength of our study is the representativity of the T1D population from the sample. Our study reports age (38.8 + 12.8years), BMI (25.2 + 4.2kg/m2), T1D evolution (19.1 ± 11.7years) and mean HbA1c (7.6 ± 1.1%[60 ± 12mmol/mol]), in concordance with other epidemiological studies in Spain [28, 29] and other countries [21, 26].

One limitation of this SED1 study is related to the cross-sectional study design, with no longitudinal data obtained that does not allow to determine the cause and effect. As a second limitation, the IPAQ scale is a self-report questionnaire, which may have led to overreporting of PA by participants with low capacity for PA. Finally, and also related to the IPAQ questionnaire itself, it is related to the validity of IPAQ in the elderly (age 65 and older), which has not yet been determined. However, this population group represented less than 4% of the sample.

In conclusion, the Spanish T1D population performed PA in a higher frequency and intensity than the general population and accomplished with general clinical recommendations. Nevertheless, a great interindividual variability is present. A relationship between PA and overall glycemic control could not be shown. However, the study's limitations should be kept in mind to discard a long-term positive influence. Some results offer opportunities to improve, such as the equity between sex in PA performance and the leisure time deserved for sports.

## Supplementary Information

**Additional file 1.**

## Data Availability

The datasets used and/or analysed during the current study are available from the corresponding author on reasonable request.

## Abbreviations

ADA: American Diabetes Association
AEMPS: Spanish Agency of Medicines and Medical Devices
BMI: Body mass index
CGM: Continuous glucose monitoring
CV: Cardiovascular
CSII: Continuous subcutaneous insulin infusion
DM: Diabetes mellitus
EASD: European Association for the Study of Diabetes
EC: Ethics Committee
HbA1c: Glycated haemoglobin A1c
IPAQ: International Physical Activity Questionnaire
MET: Metabolic equivalent
PA: Physical activity
SD: Standard deviation
SED: Spanish Diabetes Society
SMBG: Self-monitoring of blood glucose
T1D: Type 1 diabetes mellitus
T2D: Type 2 diabetes mellitus
