# Supplementary material for: Physical activity patterns in type 1 diabetes in Spain: The SED1 study
Source: BMC Sports Sci Med Rehabil. 2023 Jul 26;15:92. doi: 10.1186/s13102-023-00695-3 (PMC10369829; doi:10.1186/s13102-023-00695-3)
Supplement: Supplementary file 1 — Additional file 1. [file 13102_2023_695_MOESM1_ESM.docx]

**Supplementary Material**

**Table 1S. Insulin treatment management characteristics of the studied population**

|  | Población total  N=592 |
| --- | --- |
| Administration Method , n (%)  Basal Bolus  Premixed insulin  Continuous subcutaneous insulin infusion (CSII) | 453 (77.7%)  9 (1.5%)  121 (20.8%) |
| Total daily insulin dose (UI/kg/day), mean (DE) | 0.6 (0.3) |
| Basal Insuline Dose (UI/kg/day), mean (SD) | 02 (0.2) |
| Fast Acting Insuline Dose (UI/kg/ day ), mean (SD) | 0.3 (0.2) |
| Use of CGM/SMBG , n (%)  SMBG  CGM | 458 (77.5%)  133 (2.5%) |
| Use of advanced insulin therapy , n (%)  No  Yes | 530 (89.5%)  62 (10.5%) |
| Presence of Hipoglycemia, n (%)  No  Yes | 123 (21.1%)  460 (78.9%) |
| Visits to healthcare professionals related to T1D in the 12 months prior to the visit n (%)  No  Yes | 33 (5.6%)  559 (94.4%) |
| Hospitalization related to T1D in the 12 months prior to the visit n (%)  No  Yes | 561 (94.8%)  31 (5.2%) |

SD: standard deviation; SMBG: self-monitoring blood glucose; CMG: Continuous glucose monitoring;

**Table 2S. Physical activity and T1D treatment, sociodemographical, or clinical variables associations**

|  | **Total Walking**  **MET min/week** | **Total Moderate**  **MET min/week** | **Total Vigorous**  **MET min/week** | **Total**  **MET min/week** |
| --- | --- | --- | --- | --- |
| Diabetes therapeutic education | 0.8129 | 0.5802 | 0.7965 | 0.6683 |
| HbA1c | 0.6307 | **0.0293** | 0.0761 | **0.0454** |
| Preprandial glucose * | 0.7018 | 0.4777 | 0.5240 | 0.5914 |
| Postprandial glucose* | 0.1167 | 0.7046 | 0.1017 | 0.1866 |
| Hypoglucemya | 0.2579 | 0.1801 | 0.1070 | 0.4264 |
| CV risk | 0.0861 | 0.8572 | 0.7687 | 0.4452 |
| Method of insulin administration* | 0,3535 | 0,3196 | 0,3425 | 0,5183 |
| Total dose of insulin* | 0.5608 | 0.6229 | 0.6039 | 0.5033 |
| Rapid -acting insulin dose* | 0.9758 | 0.9505 | 0.3520 | 0.5830 |
| Long -acting insulin dose* | 0.4426 | 0.5720 | 0.1212 | 0.1689 |
| Visits to healthcare providers in the last 12 months | 0.3617 | 0.6995 | 0.5858 | 0.6561 |
| Hospital admissions | 0.5548 | 0.0583 | 0.4433 | 0.6602 |

p-adjusted by age (continuous variable), sex, BMI (continuous variable) and T1D duration, adjusted for the other variables (It has been adjusted excluding the observed variable.)

*p-value without being adjusted.

CV: Cardiovascular
